# Environmental and nutritional determinants of non-infectious pig diseases in Armenia: An ecology-based multi-regional cross-sectional study

**DOI:** 10.14202/vetworld.2026.1470-1483

**Published:** 2026-04-24

**Authors:** Zhanna Melkonyan, Valeri Grigoryan, Spartak Yeribekyan, Anahit Manvelyan, Astghik Pepoyan, Vardan Tsaturyan, Liana Grigoryan

**Affiliations:** 1 Research Center for Veterinary Medicine and Veterinary Sanitary Expertise, Armenian National Agrarian University, Yerevan, Armenia; 2 Department of Food Safety and Biotechnology, Armenian National Agrarian University, Yerevan, Armenia; 3 International Association for Human and Animal Health Improvement, Yerevan, Armenia; 4 Military Medical Faculty, Yerevan State Medical University, Yerevan, Armenia

**Keywords:** agro-climatic factors, Armenia, mycotoxins, non-infectious diseases, pigs, selenium deficiency, vitamin D deficiency, swine health

## Abstract

**Background and Aim:**

Non-infectious diseases pose a significant but often underrecognized challenge to pig production in many regions where environmental variability and nutritional issues interact. Armenia offers a unique ecological setting for studying these diseases due to its pronounced agro-climatic diversity, including steep elevation gradients, varying sunlight exposure, and region-specific soil mineral deficiencies. These environmental factors may increase the risk of metabolic imbalances, digestive problems, and toxicoses under typical farm conditions. This study aimed to assess the prevalence, regional distribution, and ecological factors driving non-infectious diseases in pigs across Armenia’s major pig-producing areas. It also explored how agro-climatic conditions, nutritional deficiencies, and feed-related mycotoxin exposure jointly influence disease patterns at the population level.

**Materials and Methods:**

A cross-sectional observational study was carried out between 2023 and 2024 on 15 pig farms across four major Armenian pig-producing regions: Ararat, Armavir, Kotayk, and Syunik. A total of 3,370 pigs from various production categories were clinically examined. These assessments focused on non-infectious conditions such as metabolic disorders, digestive diseases, toxicoses, anemia, and musculoskeletal abnormalities, following standardized veterinary diagnostic guidelines. Blood samples from 40 pigs (10 from each region) were analyzed for hematological and biochemical indicators of metabolic and mineral status, including calcium (Ca), phosphorus (P), selenium (Se), and vitamin D levels. Feed samples from each farm were evaluated to determine their basic nutritional composition and screened for major mycotoxins, such as aflatoxins, zearalenone, and deoxynivalenol. The statistical analysis incorporated descriptive statistics, Pearson chi-square tests, correlation analysis, and exploratory ecological regression to explore relationships between environmental factors and disease prevalence.

**Results:**

Overall, 825 pigs (24.4%) showed signs of non-infectious diseases. Regional prevalence ranged from 20.0% in lowland areas to 33.7% in high-altitude regions. Metabolic disorders and toxicoses made up about 30% of cases, followed by digestive diseases (20%), with anemia, musculoskeletal disorders, and mineral imbalance–related neurological syndromes each accounting for around 10%. High-altitude regions (Kotayk and Syunik) showed significant biochemical deficiencies, including decreases in serum vitamin D (45%–60%), Ca (20%–30%), and Se (40%–60%) compared to normal reference ranges. There were strong negative correlations between altitude and serum vitamin D levels (r = −0.76), as well as Ca–P balance (r = −0.67 to −0.72). Hematological data indicated anemia, inflammatory responses, and impaired protein metabolism in areas with greater environmental stress. Feed analysis uncovered widespread subclinical co-contamination with multiple mycotoxins, especially aflatoxins, zearalenone, and deoxynivalenol, suggesting chronic metabolic stress even though toxin levels remained below regulatory limits.

**Conclusion:**

Non-infectious pig diseases in Armenia mainly stem from the interaction of environmental and nutritional factors rather than isolated risks. Reduced sunlight exposure at high altitudes, along with resulting vitamin D deficiency, mineral imbalances, and chronic low-level mycotoxin exposure, create biological pathways that contribute to disease development. These findings emphasize the need for region-specific preventive measures, such as targeted vitamin D and mineral supplements in high-altitude farming systems and improved feed quality monitoring to reduce chronic mycotoxin exposure. Combining agro-climatic data with physiological diagnostics may enhance early risk detection and enable more accurate management strategies for pig health in diverse environmental production systems.

## INTRODUCTION

Pig production is highly sensitive to environmental conditions, feed quality, and husbandry practices, making non-infectious diseases a major constraint to productivity in many regions of the world [1, 2]. Pigs are especially vulnerable to metabolic imbalances, digestive disorders, and toxicoses in agro-climatic environments where climatic variability, mineral-deficient soils, and fluctuating nutritional inputs intersect [3–5]. These issues often develop gradually, remain clinically silent in early stages, and eventually lead to impaired growth, reproductive losses, and decreased herd efficiency, creating challenges for both veterinary diagnostics and preventive management [6].

Armenia presents a unique ecological environment for studying non-infectious pig diseases because of its significant agro-climatic diversity. Steep elevation changes, region-specific soil mineral deficiencies, and notable differences in sunlight exposure create different physiological stresses on pig populations across the country’s main production zones [7, 8]. Lowland areas are more often affected by variations in feed quality and storage issues, while high-altitude regions are marked by less ultraviolet radiation, limited vitamin D production, and disrupted mineral metabolism [9]. These environmental factors, combined with management differences, contribute to distinct regional patterns of metabolic disorders, digestive issues, and toxicoses in real farming conditions.

Although international studies indicate that climatic stress, micronutrient imbalance, and feed-borne contaminants often act synergistically to compromise metabolic stability in pigs [10, 11], such interacting factors have not been systematically evaluated across Armenia’s diverse agro-climatic regions. Existing observations remain fragmented and largely descriptive, which limits the understanding of how ecological exposures lead to measurable physiological disturbances and clinical disease at the population level.

Despite growing international recognition that climatic stress, micronutrient imbalance, and feed-borne contaminants interact to influence metabolic health in pigs, these factors have rarely been evaluated together under real farm conditions across ecologically diverse regions. Most previous studies have examined individual determinants, such as nutritional deficiency, climatic stress, or mycotoxin exposure, in controlled experimental settings or within relatively homogeneous production environments. Consequently, the combined influence of agro-climatic variability, mineral-deficient soils, sunlight availability, and feed safety on the epidemiology of non-infectious diseases in pigs remains poorly understood at the population level. In Armenia, available information on non-infectious pig diseases is largely fragmented and descriptive, with limited integration of clinical findings, laboratory diagnostics, environmental variables, and feed-related risk factors. Furthermore, the mechanisms by which altitude-related environmental constraints, impaired vitamin D synthesis, mineral imbalance, and chronic exposure to feed-borne mycotoxins interact to influence disease patterns have not been systematically investigated. This lack of integrated ecological–physiological assessment hampers the development of evidence-based preventive strategies tailored to regional production systems and environmental conditions.

The present study was designed to conduct a multi-regional, ecology-based investigation of non-infectious diseases in pigs across major pig-producing regions of Armenia. Specifically, the study aimed to determine the prevalence and distribution of non-infectious disease categories in pigs and to evaluate the relationships between agro-climatic conditions, nutritional factors, and feed-related contaminants under routine farm conditions. By combining clinical examination with hematological and biochemical indicators, environmental variables, and feed safety assessments, the study sought to identify biologically plausible pathways linking ecological exposure to physiological disturbances and clinical disease. Particular emphasis was placed on evaluating the roles of altitude-related sunlight limitation, reduced vitamin D availability, mineral imbalances involving Ca, P, and Se, and chronic exposure to feed-associated mycotoxins in shaping regional disease patterns. Through this integrated ecological approach, the study aimed to generate evidence that could support the development of region-specific preventive strategies and improve herd health management in pig production systems operating under diverse environmental conditions.

## MATERIALS AND METHODS

### Ethical approval

This study was conducted in accordance with internationally accepted ethical standards for research involving animals and was approved by the Ethics Committee of the Armenian National Agrarian University and the Higher Education and Science Committee of the Republic of Armenia under protocol number 10-15-21AG (approved on October 21, 2021). The approval covers a long-term institutional research program investigating metabolic health, nutritional disorders, and non-infectious diseases in farm animals in Armenia, within which the present study was implemented during the 2023–2024 field investigation period. All procedures performed during farm visits were carried out in compliance with national veterinary legislation of the Republic of Armenia and institutional guidelines governing the ethical use and handling of animals in scientific research.

The study was observational in nature and was conducted under routine farm management conditions without applying experimental treatments, invasive interventions, or procedures that could cause harm or distress to the animals. Clinical examinations were performed by a licensed veterinarian using standard veterinary diagnostic practices recommended by the World Organization for Animal Health. Animals were observed for general health status, locomotion, body condition, and signs of metabolic, digestive, hematological, or musculoskeletal disorders. The examinations were performed in a manner designed to minimize stress and avoid disruption of normal farm routines.

Blood sampling for hematological and biochemical analyses was conducted on a limited subset of animals using standard veterinary clinical procedures. Samples were collected by trained veterinary personnel using sterile techniques and appropriate animal restraint to ensure both animal welfare and sample integrity. The number of animals sampled was restricted to the minimum required to obtain reliable physiological indicators of mineral and metabolic status, thereby following the principle of reduction in animal experimentation.

All participating pig farms voluntarily agreed to take part in the study. Prior to clinical examinations and sampling procedures, farm owners or managers were informed about the purpose of the research, the procedures to be performed, and the intended use of the data. Written informed consent was obtained from farm owners allowing clinical assessment of animals and the collection of anonymized data and biological samples for scientific analysis.

Throughout the investigation, the welfare of animals was prioritized. Animals showing signs of acute infectious disease or severe clinical conditions requiring immediate treatment were excluded from the research dataset and were managed according to routine veterinary care practices on the respective farms. The study adhered to the principles of responsible animal research, including the 3Rs framework (Replacement, Reduction, and Refinement), ensuring that animal involvement was minimized and that all procedures were conducted using refined veterinary techniques to reduce discomfort and stress.

### Study period and location

A cross-sectional observational epidemiological study was carried out from January 2023 to December 2024 to examine the incidence of non-infectious diseases in pigs across selected agro-climatic regions of Armenia. The cross-sectional design was selected to assess the distribution of clinical conditions along with related environmental and nutritional factors under routine farm conditions.

Farm visits and sampling took place during the summer seasons of 2023–2024 to reduce seasonal differences in sunlight exposure and feed storage conditions, which are crucial for understanding vitamin D status and mycotoxin exposure.

### Agro-climatic characteristics

The study was carried out in four key Armenian pig-producing regions: Ararat, Armavir, Kotayk, and Syunik. These areas were chosen to represent the main pig farming zones in the country and to reflect a significant agro-climatic gradient in terms of altitude, sunlight exposure, temperature, and soil mineral composition.

Ararat and Armavir are lowland areas with higher annual sunlight, warmer temperatures, and issues related to feed storage and heat stress. Kotayk and Syunik are high-altitude areas with less sunlight, cooler weather, and well-documented soil micronutrient deficiencies, especially Se and Ca.

Environmental variables such as regional altitude, annual average temperature, total yearly precipitation, and number of sunny days were collected from official national hydrometeorological and agricultural reports. Instead of farm-specific data, long-term regional averages were used, and all environmental information was analyzed at the regional (ecological) level.

### Farm selection criteria

Pig farms were chosen through purposive sampling to represent semi-intensive and commercial production systems common to each study region. The inclusion criteria included active pig production during the study period and voluntary consent from farm owners to participate in clinical examinations and sample collection. No minimum herd size was set.

All farms approached agreed to participate in the study, and no refusals were recorded. Each farm was visited once during routine veterinary inspections. The selection process was designed to ensure representation of the main production systems within each region, rather than to achieve statistically random sampling at the national level, which is a limitation inherent to ecological field studies.

### Farms and animals

Purposive sampling was used to select a total of 15 pig farms representing semi-intensive and commercial production systems typical of the studied regions. Farms varied in herd size, feeding practices, and housing conditions. All animals present on each farm at the time of the visit were included in the assessment, resulting in a census-like evaluation of 3,370 pigs.

The animals examined spanned different age and production groups, including piglets, growers, and sows. Growers were grouped with piglets for statistical analysis because both are growing animals and have similar physiological susceptibility to nutritional and metabolic issues. All pigs were commercial crossbreeds originating from English Yorkshire and Landrace lines (DanBred-type hybrids). Sex distribution was not systematically recorded.

Animals were mainly kept indoors on concrete-slatted floors, with automated ventilation systems used to control the microclimate across farms.

Supplementary Table S1 summarizes detailed farm-level characteristics, including herd size, housing system, feeding system, and the number of clinically affected pigs.

### Clinical assessment

All pigs were examined clinically by the same veterinarian to ensure consistent assessment. The clinical evaluation focused on non-infectious conditions, including metabolic disorders, digestive disturbances, toxicoses, musculoskeletal abnormalities, anemia, and related syndromes, following the guidelines of the World Organization for Animal Health (OIE) [12, 13].

The clinical examination involved assessing general appearance and behavior, locomotion and posture, body condition and growth, skin and mucous membranes, hydration status, and capillary refill time. Special attention was paid to signs of metabolic imbalance (e.g., bone deformities, tetany, and muscle weakness), digestive disorders (e.g., diarrhea, dyspepsia, and atony), toxicoses (e.g., anorexia, neurological signs, and hemorrhagic manifestations), and musculoskeletal abnormalities (e.g., lameness and joint swelling).

Animals exhibiting clinical signs indicative of acute or chronic infectious diseases were excluded from the non-infectious disease dataset and were not part of the statistical analyses.

Farm documentation was reviewed to gather information on feeding routines, previous illnesses, and routine veterinary interventions.

Overlapping pathological conditions were categorized using a comprehensive etiological approach. For each case, a primary etiological diagnosis (e.g., alimentary disorder or toxicosis) was assigned based on the main causal factor, followed by documentation of the leading clinical syndrome (e.g., rickets, alimentary anemia, or mycotoxic hepatopathy). In cases of combined etiology (e.g., nutrient deficiency occurring alongside chronic mycotoxin exposure), conditions were classified as complex, with both primary and contributing factors specified.

### Hematological and biochemical analyses

Blood samples were collected from animals that were clinically examined during routine farm visits. Sampling took place in the summer mornings while animals were fasting. A total of 10 pigs were sampled from each region, resulting in 40 blood samples.

Hematological analysis included hemoglobin concentration, hematocrit, erythrocyte count, leukocyte count, and erythrocyte sedimentation rate (ESR). Tests were performed using automated veterinary hematology analyzers based on impedance and photometric principles, following the manufacturer’s instructions and standard veterinary laboratory protocols.

Biochemical analysis focused on indicators of mineral and metabolic status, including serum Ca, P, Se, and vitamin D. Serum Ca and P concentrations were measured using colorimetric methods, while serum Se and vitamin D levels were determined using enzyme-linked immunosorbent assay (ELISA)-based veterinary diagnostic kits. All measurements were expressed in standardized units.

Hematological and biochemical results were interpreted using established physiological reference ranges for swine from veterinary clinical standards and laboratory guidelines.

All laboratory analyses were conducted in certified veterinary diagnostic laboratories using validated protocols and commercially available diagnostic kits. Internal quality control procedures were routinely implemented to ensure analytical accuracy and reproducibility.

### Feed evaluation and screening for mycotoxins

Feed samples were collected from each farm during routine visits to assess nutritional characteristics and check for potential mycotoxin contamination. Basic feed composition, including moisture, crude protein, and fiber content, was evaluated to give a general picture of dietary quality in field conditions.

Mycotoxin screening focused on major contaminants relevant to swine, including aflatoxins, zearalenone, and trichothecenes, especially deoxynivalenol (DON). Analyses were conducted using ELISA-based assays commonly used in veterinary feed safety monitoring. The detection limits ranged from 0.1 to 5 μg/kg, while the quantification limits ranged from 1 to 10 μg/kg depending on the specific mycotoxin.

Laboratory analysis showed a high occurrence of mycotoxins appearing together within individual feed samples. The frequent detection of aflatoxin B1 and zearalenone was observed in samples from the Ararat region. The most complex contamination patterns, including aflatoxins, zearalenone, and DON, were found in feed samples from the Kotayk region, which also showed more noticeable clinical signs of metabolic and immune-related issues in pigs.

Detected mycotoxin levels were evaluated based on internationally accepted guidelines for contaminants in pig feed. Considering the chronic and multifactorial nature of exposure in field conditions, mycotoxin results were combined with clinical observations and biochemical indicators to support regional interpretation of non-infectious disease patterns rather than to determine acute toxicity.

### Classification of non-infectious diseases

Final diagnoses were made by integrating clinical examination findings, hematological and biochemical results, and feed analysis data. Disease conditions were classified into metabolic disorders, digestive diseases, toxicoses, anemia and hematological dysfunctions, musculoskeletal disorders, and mineral imbalance–related neurological syndromes, following the classification frameworks of the OIE and the Merck Veterinary Manual [12-14].

### Descriptive and inferential statistics

Descriptive statistics were used to determine disease category prevalence across different regions and age groups. Differences in categorical disease distribution between piglets and sows were evaluated using Pearson’s chi-square test. Prevalence estimates were provided with 95% confidence intervals (CI) calculated through binomial proportion methods.

Regional differences in hematological and biochemical parameters were assessed using Student’s t-test, with a significance level set at p < 0.05. Prior to parametric testing, the data distribution was visually examined for approximate normality and homogeneity of variance.

### Multivariate and ecological analysis

Given the aggregated structure of the regional data, an exploratory ecological analysis was conducted. Pearson correlation coefficients were calculated to examine relationships between environmental factors (altitude, temperature, and sunlight exposure) and regional averages of disease prevalence and biochemical indicators (vitamin D, Ca, and P).

An ordinary least squares linear regression model was built using the overall prevalence of non-infectious diseases as the dependent variable, with altitude, mean annual temperature, and number of sunny days as predictors. Due to the small number of ecological units (n = 4 regions), regression coefficients, p-values, and model fit statistics (R², adjusted R²) are provided descriptively to show directional trends and are not interpreted as evidence of causality. All statistical analyses were performed using Python 3.10.

## RESULTS

### Overall clinical status of the examined animals

A total of 3,370 pigs from 15 farms across four agro-climatic zones of Armenia were examined clinically. Among these animals, 825 pigs were diagnosed with non-infectious diseases, representing an overall prevalence of 24.5% (95% CI: 22.9%–25.9%) (Table 1). Non-infectious conditions were found in all regions studied, indicating their widespread presence under typical farm conditions.

**Table 1:** Distribution of animals according to clinical condition.

Animal group	Total examined	Clinically healthy (heads/%)	Signs of disease (heads/%)
Piglets	2460	1845 (75.0)	615 (25.0)
Sows	910	700 (76.9)	210 (23.1)
Total	3370	2545 (75.5)	825 (24.5)

Regional prevalence varied significantly, ranging from 20.0% (95% CI: 16.8%–23.5%) in Ararat to 33.7% (95% CI: 30.6%–36.9%) in Kotayk. Detailed regional prevalence estimates with CI are included in Supplementary Table S2, and farm-level distribution data are summarized in Supplementary Table S1.

Growing animals (piglets and growers) made up the majority of affected individuals, reflecting their greater physiological sensitivity to nutritional and metabolic disturbances.

### Structure of the categories of non-infectious diseases

Among the 825 pigs diagnosed with non-infectious diseases, metabolic disorders and toxicoses were the most common categories, making up about 30% of all cases. Digestive diseases accounted for roughly 20%, while anemia, musculoskeletal disorders, and mineral imbalance–related neurological syndromes each represented about 10%.

**Figure 1:**
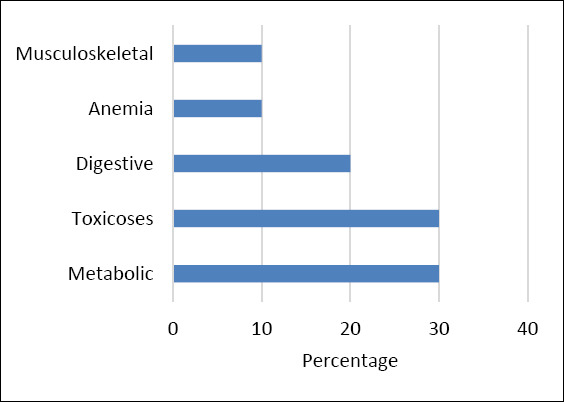
Distribution of non-infectious disease categories among clinically affected pigs (n = 825). Values indicate the proportion of each disease category among pigs diagnosed with non-infectious conditions across all regions under study.

Figure 1 shows the proportional distribution of disease categories, while Supplementary Table S2 summarizes region-specific prevalence estimates.

### Distribution of diseases by age group

Age group stratification showed a similar distribution of disease categories between piglets/growers and sows. A Pearson chi-square test comparing disease patterns in piglets (n = 615) and sows (n = 210) revealed no significant difference (χ² = 0.0003, df = 4, p = 0.99), suggesting that the overall pattern of non-infectious diseases is comparable across different ages.

Although the proportional structure was similar, the clinical presentation of the disease varied by age. Piglets and growers more often showed signs of digestive issues (e.g., dyspepsia) and early metabolic imbalance symptoms, while sows more frequently displayed neurological signs associated with mineral imbalance.

Pronounced regional differences were observed in the prevalence and main structure of non-infectious diseases across the four agro-climatic zones (Table 2).

**Table 2:** Natural and climatic conditions of Armenia’s pig-producing regions.

Region	Altitude (m)	Average annual temperature (°C)	Precipitation (mm/year)	Number of sunny days (per year)	Main soil mineral deficiencies	Risks typical of pig farming
Ararat	800–1000	+10 to +12	200–250	270–300	Selenium, zinc	Heat stress during summer and soil salinity
Armavir	850–1100	+11 to +13	250–300	250–280	Selenium, copper	Hot dry winds and limited drinking water availability
Kotayk	1200–1800	+6 to +8	500–700	160–180	Selenium, iodine, and cobalt	Vitamin D deficiency and marked temperature fluctuations
Syunik	1500–2200	+5 to +8	400–600	170–190	Selenium, calcium	Strong winds and limited pasture availability

### Regional variation in the incidence of non-infectious diseases

The lowland regions (Ararat and Armavir) showed a higher occurrence of digestive disorders and toxicoses. These patterns aligned with feed-related issues, such as fluctuations in feed quality and evidence of mycotoxin contamination, especially zearalenone and aflatoxins in Armavir.

In contrast, high-altitude regions like Kotayk and Syunik showed a predominance of metabolic disorders, including rickets, osteodystrophy, hypovitaminosis, and tetany. These areas are marked by less sunlight exposure and documented soil micronutrient deficiencies, which were reflected in consistent biochemical changes.

Although disease patterns are similar across age groups, the main causes vary by region, with feed-related issues common in lowland farms and environmentally driven metabolic problems more prevalent in high-altitude areas.

### Hematological findings

Hematological analysis showed distinct region-specific differences from standard reference ranges (Table 3). Hemoglobin and hematocrit levels decreased in all regions, with the most significant drops seen in Kotayk and Syunik, where hemoglobin ranged from 85–110 g/L and hematocrit from 30%–39%. These results align with nutritional anemia.

**Table 3:** Hematological parameters of pigs across agro-climatic regions and possible contributing factors.

Parameter	Normal range	Ararat	Armavir	Kotayk	Syunik	Possible contributing factors
Hemoglobin (g/L)	100–140	98–120 ↓	95–113 ↓	85–110 ↓↓	88–115 ↓↓	Nutritional insufficiency, mineral absorption impairment, and chronic metabolic stress
Hematocrit (%)	36–43	35–41	34–40 ↓	30–38 ↓↓	32–39 ↓	Reduced erythropoiesis, mineral imbalance, and chronic physiological stress
Leukocytes (×10⁹/L)	8.0–16.0	15–18 ↑	14–17 ↑	17–21 ↑↑	16–20 ↑↑	Stress-related and inflammatory responses
Erythrocytes (×10⁹/L)	6.0–7.5	6.2–7.1	6.0–7.0 ↓	5.5–6.8 ↓	5.8–6.9 ↓	Micronutrient insufficiency and metabolic disturbance
Total protein content (g/L)	70–85	68–82	65–80 ↓	60–78 ↓↓	63–79 ↓	Impaired protein metabolism, limited feed quality, and chronic metabolic stress
ESR (mm/h)	20–35	30–40 ↑	23–38 ↑	35–45 ↑↑	32–42 ↑↑	Chronic inflammation or stress-related responses

↑ and ↓ indicate mild deviations from physiological reference ranges; ↑↑ and ↓↓ indicate marked deviations.

White blood cell counts surpassed the upper normal limit in all areas, with the highest readings in Kotayk (17–21 × 10^9^/L) and Syunik (16–20 × 10^9^/L), suggesting ongoing inflammatory or stress responses.

Erythrocyte counts were somewhat lower in high-altitude regions, and total serum protein levels were lowest in Kotayk and Syunik (60–78 g/L). The ESR was elevated in all regions, reaching up to 45 mm/h in high-altitude areas.

Overall, the hematological profiles reflect a combination of anemia, inflammatory response, and disrupted protein metabolism in areas with significant environmental and nutritional challenges.

### Biochemical alterations

Feed analysis revealed region-specific patterns of mycotoxin contamination, while biochemical parameters showed consistent deviations from reference ranges (Table 4). Although the measured concentrations stayed below internationally accepted regulatory limits, the frequent co-occurrence of multiple mycotoxins was observed.

**Table 4:** Biochemical blood parameters of pigs by region (2023–2024).

Parameter	Normal range	Ararat	Armavir	Kotayk	Syunik	Possible contributing factors
Calcium (mmol/L)	2.5–3.0	2.1–2.4 ↓	2.3–2.5	1.9–2.1 ↓	2.0–2.3 ↓	Reduced vitamin D availability, regional soil mineral limitations, and high-altitude conditions
Phosphorus (mmol/L)	1.8–2.5	1.5–1.8 ↓	1.6–2.0 ↓	1.2–1.5 ↓	1.3–1.7 ↓	Dietary imbalance and reduced bioavailability of minerals
Selenium (μg/mL)	0.07–0.15	0.05–0.07 ↓	0.04–0.06 ↓	0.03–0.05 ↓	0.06–0.08 ↓	Low selenium availability in regions
Vitamin D (ng/mL)	30–38	18–25 ↓	22–28 ↓	12–19 ↓	15–20 ↓	Limited exposure to sunlight and predominantly indoor housing

↓ indicates values below the physiological reference range.

Ararat was mainly linked to aflatoxin contamination, Armavir exhibited higher levels of zearalenone, and Kotayk and Syunik showed more frequent detection of DON. Co-exposure to multiple mycotoxins, especially in Armavir, Kotayk, and Syunik, was common.

These contamination patterns were linked with lower serum protein levels, higher leukocyte counts, and increased rates of toxicoses and digestive disorders, indicating that long-term, subclinical exposure to mycotoxins may lead to metabolic and inflammatory stress.

### Feed quality and mycotoxin contamination

Feed quality assessment showed frequent subclinical contamination with multiple mycotoxins in all regions studied. Aflatoxins, zearalenone, and DON were commonly found, often together, although the concentrations generally stayed below regulatory limits.

Distinct regional contamination profiles were identified. In lowland areas, aflatoxins and zearalenone were dominant, while DON was more commonly found in high-altitude regions. The most complex co-contamination patterns, involving aflatoxins, zearalenone, and DON, occurred in the Kotayk region.

Overall, these findings show that chronic, low-level mycotoxin co-exposure was common under routine farm conditions and was a consistent background feature of the production systems studied.

### Integrated interpretation of environmental, nutritional, and clinical indicators

**Figure 2:**
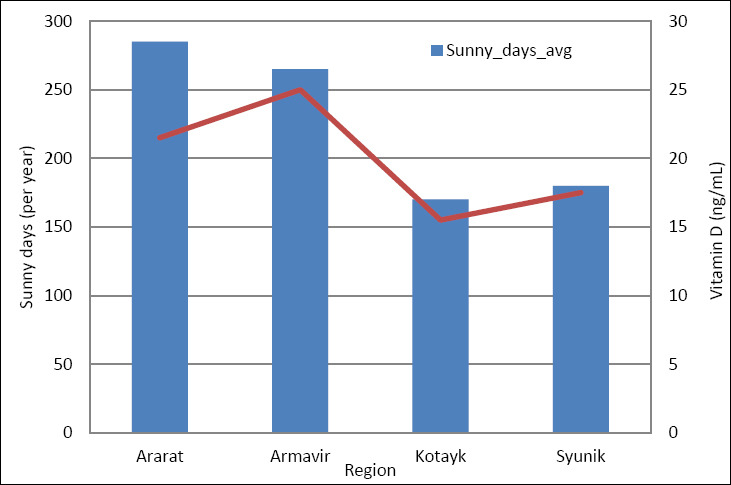
Relationship between sunlight exposure (number of sunny days per year) and serum vitamin D levels across Armenian pig-producing regions (n = 4 regions; 10 pigs sampled per region). Points represent regional mean vitamin D concentrations; the line indicates the association direction based on Pearson correlation analysis.

Strong links were seen between environmental factors and the prevalence of non-infectious diseases at the regional level (Figure 2). Altitude showed a strong negative correlation with serum vitamin D levels (r = −0.76) and with Ca–P balance (r = −0.67 to −0.72).

**Figure 3:**
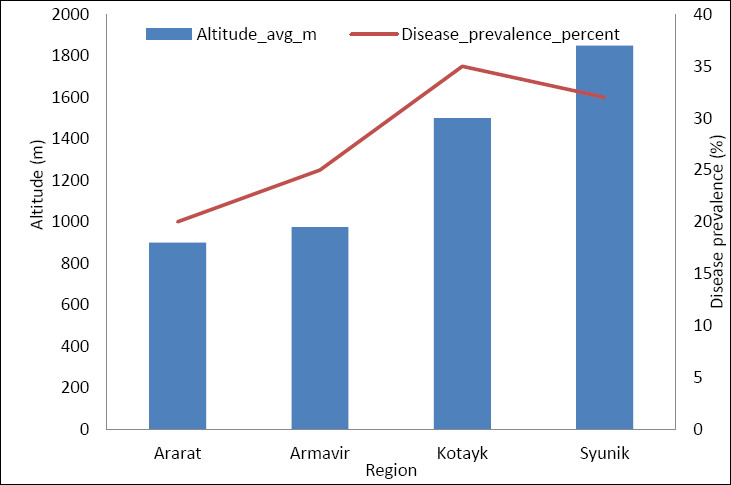
Relationship between altitude and prevalence of non-infectious diseases across Armenian pig-producing regions (n = 4 regions) Each point represents a regional aggregate, and the fitted line reflects an exploratory linear regression illustrating the directional trend.

Exploratory ecological regression analysis identified altitude as a significant regional predictor of non-infectious disease prevalence (Figure 3). Supplementary Table S3 shows the regression coefficients and model fit statistics. Because of the small number of ecological units (n = 4 regions), these results are considered exploratory and suggestive of directional trends rather than definitive causal effects.

Given the dataset’s aggregated regional structure (n = 4 regions), regression analyses were considered exploratory and descriptive. The model fit was evaluated qualitatively to identify directional trends and ecological coherence rather than to produce precise inferential estimates. Therefore, numerical R² values and CI were not emphasized to avoid overstating statistical precision within the limited ecological unit constraints.

**Figure 4:**
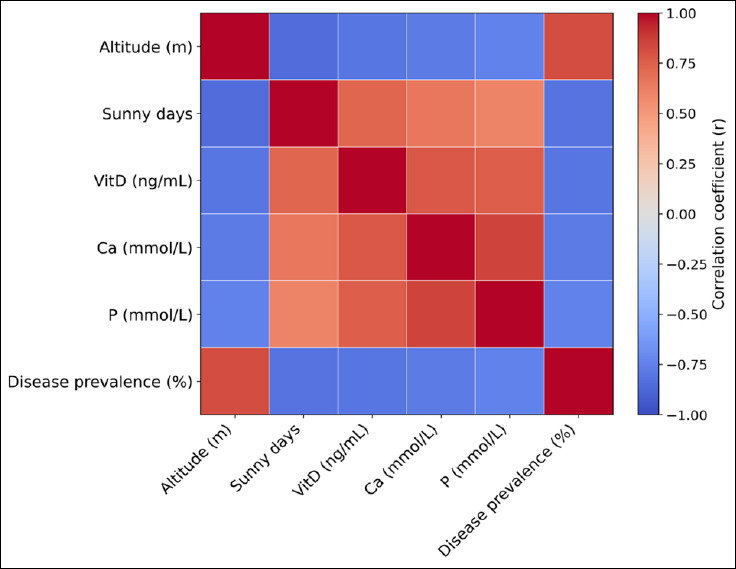
Correlation heatmap illustrating associations between agro-climatic variables, biochemical indicators, and prevalence of non-infectious diseases across regions. Correlations are based on aggregated regional means (n = 4 regions) and represent ecological associations rather than individual-level relationships.

High-altitude regions consistently showed the most severe biochemical deviations and the highest rates of metabolic disorders, while lowland regions had higher frequencies of feed-related toxicoses and digestive issues. These findings show that non-infectious pig diseases in Armenia result from interacting agro-climatic and nutritional factors, forming integrated ecological–physiological pathways instead of isolated risk factors (Figure 4).

## DISCUSSION

### Integrated ecological interpretation of the findings

This study offers a multi-regional, ecology-based assessment of non-infectious pig diseases in Armenia by combining clinical, hematological, biochemical, environmental, and feed-related indicators under routine farm conditions. Unlike traditional prevalence surveys that report disease occurrence in isolation, the findings suggest that non-infectious pig diseases are influenced by interacting agro-climatic and nutritional factors. The regional patterns observed support biologically coherent ecological–physiological pathways through which environmental exposure leads to physiological disruption and, ultimately, clinical disease manifestation.

Although this cross-sectional design does not define specific supplementation doses or intervention intervals, the findings support routine seasonal monitoring of serum vitamin D, Ca, P, and Se, particularly in high-altitude indoor production systems, to facilitate evidence-based preventive decisions. At the herd and policy levels, the results advocate for the development of regionally adapted preventive frameworks for pig production in Armenia, including targeted micronutrient supplementation, enhanced feed safety surveillance, and veterinary prevention strategies aligned with local agro-climatic conditions.

### Environmental and nutritional pathways associated with disease development

One of the key findings of this study is the strong link between altitude-related environmental factors and the burden of metabolic disease in pigs. High-altitude areas, especially Kotayk and Syunik, consistently showed lower serum levels of vitamin D, Ca, P, and Se concentrations, together with higher rates of rickets, osteodystrophy, tetany, and growth retardation. These findings support a biologically plausible pathway where reduced sunlight exposure limits the body’s ability to produce vitamin D synthesis, impairs Ca–P metabolism, and leads to skeletal and neuromuscular disorders. Less exposure to ultraviolet radiation in high-altitude and low-sunlight environments has previously been associated with impaired vitamin D production and subsequent disturbances in Ca–P metabolism in pigs and other livestock species [9, 15, 16].

Previous experimental studies have confirmed the importance of vitamin D and mineral balance for bone development and metabolic stability in pigs [3, 17]. However, most available evidence has been generated under controlled feeding conditions rather than through field-based ecological assessment. The present study expands on this knowledge by showing that altitude-related hypovitaminosis D can remain a significant risk factor even in modern indoor production systems, where routine dietary supplementation is often assumed to provide sufficient protection. These findings challenge the idea that vitamin D deficiency is of limited relevance in intensively housed pig populations.

Se availability and bioaccessibility are increasingly recognized as region-dependent factors that influence antioxidant defense, metabolic stability, and disease susceptibility in livestock systems [18, 19]. The consistently low Se concentrations observed across all studied regions further highlight Armenia’s Se-poor soil profile and its likely systemic effects on pig health. Se deficiency has been linked to anemia, weakened immune function, muscular weakness, and increased vulnerability to metabolic stress [20]. The more significant Se deficits seen in high-altitude regions provide a plausible mechanistic explanation for the hematological changes observed in this study, including lower hemoglobin and hematocrit levels along with higher inflammatory markers.

### Contribution of feed safety and mycotoxin exposure

Feed safety has become a key factor influencing metabolic health in the studied production systems. Chronic exposure to low doses of mycotoxins can disrupt nutrient absorption, hormonal regulation, and immune function, even when contamination levels are below regulatory standards [11, 18, 19]. An important aspect of this study is showing that subclinical multi-mycotoxin contamination can significantly contribute to the development of non-infectious diseases, even without overt toxic concentrations. In the regions studied, feed samples often contained multiple toxins such as aflatoxins, zearalenone, and DON, a contamination pattern increasingly seen as biologically significant, despite remaining under official regulatory limits [11].

In lowland areas like Ararat and Armavir, higher temperatures and storage-related feed issues were linked to aflatoxin and zearalenone contamination. These conditions coincided with increased rates of digestive disorders and toxicoses. Conversely, DON was more common in high-altitude areas, where it may have worsened nutrient malabsorption and inflammatory responses. These observations align with increasing evidence that chronic low-level mycotoxin exposure can disrupt protein metabolism, harm intestinal integrity, and intensify metabolic stress, especially when paired with micronutrient deficiencies [10].

Taken together, these results suggest that compliance with regulatory limits does not necessarily ensure biological safety in real farm conditions. Instead, repeated exposure to multiple mycotoxins, along with environmental and nutritional stressors, may work together and contribute to the chronic disease patterns seen in Armenian pig farms.

### Hematological and biochemical indicators as markers of ecological stress

The hematological and biochemical changes documented in this study offer a comprehensive view of the physiological effects of environmental and nutritional limitations. Elevated leukocyte counts and ESR across regions indicate ongoing inflammatory or stress-related responses, while lower erythrocyte counts, hemoglobin levels, and total serum protein suggest impaired red blood cell production and altered protein metabolism.

These laboratory findings closely mirrored the regional clinical patterns, strengthening the interpretation that environmental exposure can lead to measurable physiological disruptions before or alongside clear clinical disease. While integrating biomarkers with environmental data is still underused in swine epidemiology, it offers significant value for early risk detection and targeted preventive measures.

### Ecological interpretation and caution regarding causality

The exploratory ecological regression analysis identified altitude as a key regional predictor of non-infectious disease prevalence. Although the strength of the observed associations emphasizes the likely importance of environmental gradients, the limited number of regions warrants cautious interpretation. As with all ecological studies, associations found at the regional level cannot be directly applied to individual animals.

Therefore, the current findings should be viewed as evidence of interacting factors and directional trends rather than definitive proof of causality. However, the consistency seen across clinical, biochemical, hematological, feed-related, and environmental indicators supports a coherent pathway-based interpretation linking agro-climatic conditions with disease outcomes.

### Implications for region-adapted prevention

The current findings offer a practical framework for region-specific prevention of non-infectious pig diseases. In high-altitude areas, preventive measures should focus on vitamin D and mineral supplementation tailored to limited sunlight exposure and regional soil micronutrient deficiencies. In lowland areas, enhancing feed storage practices and routine mycotoxin monitoring are particularly important for reducing digestive issues and toxicoses.

This approach encourages a move from broad management advice to more specific livestock health strategies tailored to regional risk profiles by using ecological context and physiological diagnostics together.

### Relevance beyond Armenia

Although this study was performed in Armenia, its implications extend to pig production systems in other mountainous, mineral-poor, and low-sunlight regions, including parts of the Caucasus, Central Asia, and similar highland areas worldwide. In this context, Armenia can serve as a model illustrating how agro-climatic constraints may influence the risk of metabolic disease under modern pig farming conditions. Environmental bioindicator studies have increasingly demonstrated that regional ecological stressors, such as altitude, soil mineral depletion, and climate variability, can have measurable physiological effects across species, further underscoring the importance of ecology-based approaches in livestock health assessment [21].

The novelty of this study lies in its ecology-based integration of agro-climatic exposure, feed safety, and host biochemical markers to identify region-specific pathways that lead to non-infectious pig diseases under routine farm conditions, rather than focusing on isolated risk factors. Recent advances in precision feeding and livestock management also highlight the importance of combining environmental exposure, feed quality, and physiological monitoring to decrease disease risk and enhance resilience in various production settings [22, 23].

### Study limitations

Despite the comprehensive ecological and physiological assessment conducted in this study, several limitations should be recognized. First, the cross-sectional design prevents establishing definitive temporal or causal relationships between environmental exposure, nutritional status, and disease development. Although the observed associations are biologically plausible and supported by consistent clinical, biochemical, and environmental findings, longitudinal and intervention studies are necessary to verify the timing and direction of the proposed pathways.

Second, the environmental analysis was ecological in nature, with agro-climatic variables assessed at the regional level using long-term averages rather than farm-specific or animal-specific measurements. Although this approach is suitable for identifying dominant regional patterns, it might hide within-region differences and could lead to ecological fallacy when making group-level conclusions at the individual level.

Third, although all pigs were examined by the same veterinarian using a standardized clinical protocol, some diagnostic misclassification cannot be entirely ruled out, especially for disorders with overlapping symptoms such as metabolic disturbances, subclinical toxicoses, and digestive issues. The use of an integrated etiological framework that combines clinical, hematological, biochemical, and feed analysis findings reduced this risk, but diagnostic differentiation in field conditions remains inherently difficult, particularly without molecular confirmation [24, 25].

Fourth, the study did not include molecular or culture-based analysis of the intestinal microbiota. Given the high occurrence of digestive disturbances and mycotoxin-related syndromes in various regions, microbiota composition may be an important yet unmeasured factor affecting nutrient absorption, immune regulation, and inflammatory responses [26–29]. Previous research indicates that micronutrient availability, feed quality, and microbiota-modulating strategies are closely linked in maintaining metabolic balance in livestock. Probiotic or microbial interventions could enhance gut resilience and metabolic stability under controlled conditions [30–32] and may help mitigate the physiological impacts of low-grade mycotoxins or micronutrient deficiencies in environmentally stressed systems [33–35]. However, these mechanisms were not directly examined in this study.

Fifth, feed composition data were limited to basic nutritional parameters, and detailed information on energy density, micronutrient premix formulation, and batch-to-batch variation was not consistently available across farms. Additionally, mycotoxin evaluation relied on routine screening methods commonly used in field-based veterinary monitoring, which may underestimate cumulative exposure under chronic co-occurrence conditions. However, these methods closely reflect real-world veterinary practice and therefore remain relevant [36]. Finally, other potentially important confounding factors, such as parasitic burden, water quality, subclinical viral or bacterial infections, and housing microclimate, were not directly measured and may have contributed to regional variability in disease expression [37–39]. Sex-based analyses were also not conducted because sex distribution data were incomplete at the farm-level [40, 41].

### Future research directions

Future studies should expand on these ecological findings using longitudinal designs that can capture temporal and seasonal variations, especially regarding vitamin D levels and chronic mycotoxin exposure in typical farm conditions. Controlled intervention studies assessing region-specific supplementation strategies, including vitamin D, Se, and Ca–P balancing, along with better feed management practices, are essential to strengthen causal conclusions and evaluate effectiveness at the field level. Additionally, incorporating microbiome profiling, metabolomics, and targeted probiotic interventions may help clarify the biological pathways connecting environmental factors, nutrition, and the development of non-infectious diseases in pigs, thereby aiding in the creation of more precise and region-specific prevention strategies.

## CONCLUSION

This study showed that non-infectious diseases are common in pig production systems across Armenia, with an overall prevalence of 24.4% among the examined population. The results revealed clear regional differences in disease occurrence, with higher prevalence in high-altitude regions compared to lowland areas. Metabolic disorders and toxicoses were the most common disease categories, making up about 30% of all diagnosed cases, followed by digestive disorders. Hematological and biochemical results showed consistent deviations from normal reference ranges, especially with lower levels of vitamin D, Ca, P, and Se in high-altitude regions. Additionally, feed analysis found frequent co-occurrence of multiple mycotoxins, including aflatoxins, zearalenone, and DON, even when contamination levels stayed below regulatory thresholds. These findings suggest that the burden of non-infectious pig diseases is linked to complex interactions between environmental and nutritional factors rather than single isolated causes.

From a practical standpoint, the results highlight the importance of region-specific preventive management in pig production systems. In high-altitude areas characterized by reduced sunlight exposure and soil micronutrient limitations, routine monitoring of serum vitamin D, Ca, P, and Se levels, along with targeted micronutrient supplementation, may help lower the risk of metabolic disorders and skeletal abnormalities. Conversely, lowland regions seem to require a greater focus on feed storage management and regular monitoring for mycotoxin contamination to prevent digestive disorders and toxicoses. Combining environmental factors with clinical and biochemical diagnostics offers a valuable framework for enhancing herd health management and strengthening preventive veterinary strategies across diverse agro-climatic conditions.

A key strength of this study is the integration of clinical examination, hematological and biochemical diagnostics, feed safety evaluation, and environmental indicators across multiple agro-climatic regions under real farm conditions. This ecology-based approach enables the identification of biologically coherent pathways linking environmental exposure, nutritional imbalance, and disease manifestation at the population level. By combining ecological, physiological, and management-related indicators, the study offers a comprehensive understanding of how environmental constraints lead to measurable health outcomes in pig production systems.

In conclusion, non-infectious pig diseases in Armenia seem to result from complex interactions among agro-climatic conditions, micronutrient levels, and feed safety factors. The results highlight the importance of region-specific preventive strategies that include environmental monitoring, nutritional management, and feed safety oversight. This comprehensive approach can help improve herd health, boost productivity, and create more resilient pig production systems in environmentally diverse regions.

## DATA AVAILABILITY

The supplementary data can be available from the corresponding author upon a request.

## AUTHORS’ CONTRIBUTIONS

ZM and AP: Conceived and designed the study and drafted the manuscript. ZM, VG, SY, and AM: Performed field investigations and clinical examinations. AM and VT: Conducted laboratory analyses. LG and AP: Performed data analysis and interpretation. All authors contributed to critical revision of the manuscript for important intellectual content and approved the final version.

## References

[1] Maes D G D, Dewulf J, Piñeiro C, Edwards S, Kyriazakis I (2020). A critical reflection on intensive pork production with an emphasis on animal health and welfare. J Anim Sci.

[2] Adewale C I, Ndyomugyenyi E K, Mugonola B (2024). Drivers and barriers to the choice of production systems among smallholder pig farmers:Evidence from Northern Uganda. Heliyon.

[3] Hendriks W H, Verstegen M W A, Babinszky L (2019). Poultry and pig nutrition:Challenges of the 21st century. Wageningen, Netherlands:Wageningen Academic Publishers.

[4] Manyi-Loh C, Mamphweli S, Meyer E, Okoh A Antibiotic use in agriculture and its consequential resistance in environmental sources:potential public health implications. Molecules.

[5] Alarcón L V, Allepuz A, Mateu E (2021). Biosecurity in pig farms:a review. Porc Health Manag.

[6] Augustyniak A, Pomorska-Mól M (2023). An update in knowledge of pigs as the source of zoonotic pathogens. Animals (Basel).

[7] Grigoryan V, Tspnetsyan H, Grigoryan L, Abrahamyan V, Yeribekyan Y, Petrosyan G (2025). Ixodes ticks and cases of brucellosis in Berd region of Tavush province:sustainable agriculture. Sib J Life Sci Agric.

[8] Sereda I (2025). Determining the timing of phytopathological monitoring for early detection of winter wheat diseases in the Republic of Armenia using remote sensing methods. Agr Sci Technol.

[9] Hess S S, Burns D A, Boudinot F G, Brown-Lima C, Corwin J, Foppert J D (2024). New York State Climate Impacts Assessment Chapter 05:Ecosystems. Ann N Y Acad Sci.

[10] Kovac T M, Jurcevic S (2025). New insights into mycotoxin contamination, detection, and mitigation in food and feed systems. Toxins (Basel).

[11] Akinmoladun O F, Fon F N, Nji Q, Adeniji O O, Tangni E K, Njobeh P B (2025). Multiple mycotoxin contamination in livestock feed:implications for animal health, productivity, and food safety. Toxins (Basel).

[12] OIE (2022). Terrestrial Animal Health Code.

[13] OIE (2023). Terrestrial Manual 2023.

[14] Merck Veterinary Manual (2023). Swine diseases:classification and diagnosis. Merck &Co., Inc.

[15] Barnkob L L, Petersen P M, Nielsen J P, Jakobsen J (2018). Vitamin D enhanced pork from pigs exposed to artificial UVB light in indoor facilities. Eur Food Res Technol.

[16] Kolp E, Wilkens M R, Pendl W, Eichenberger B, Liesegang A (2017). Vitamin D metabolism in growing pigs:influence of UVB irradiation and dietary vitamin D supply on calcium homeostasis, its regulation and bone metabolism. J Anim Physiol Anim Nutr (Berl).

[17] Maher S, Sweeney T, O'Doherty J V (2025). Optimising nutrition for sustainable pig production:strategies to quantify and mitigate environmental impact. Animals.

[18] Alshaal T, Domokos-Szabolcsy É, Fári M, Veres S, Kaszás L, Kovács Z (2025). Agricultural sustainability and the challenges of selenium nanoparticles (SeNPs):their role in supporting the environmental economy. Plant Stress.

[19] Oumer A, Joy E J M, De Groote H, Broadley M R, Gashu D (2024). Burden of selenium deficiency and cost-effectiveness of selenium agronomic biofortification of staple cereals in Ethiopia. Br J Nutr.

[20] Wrobel B, Zielewicz W, Paszkiewicz-Jasinska A (2025). Improving forage quality from permanent grasslands to enhance ruminant productivity. Agriculture.

[21] Fehér P, Molnár Z, Pálfi M P, PálfinéLábadi A, Plank P, Lakatos I (2025). The initial detection of mycotoxins released and accumulated in the Golden Jackal (Canis aureus):investigating the potential of carnivores as environmental bioindicators. Int J Mol Sci.

[22] Montalvo G, Rodriguez M, Piñeiro C, Calvet S, Sanz M J, Garcia-Rebollar P (2025). Precision feeding on pig fattening farms:can simplified implementation enhance productivity and reduce pollutant emissions?. Agriculture.

[23] Arun K B, Nisha P, Plessas S, Golic N, Pepoyan A (2025). Editorial:Probiotics for global health:advances, applications and challenges. Front Microbiol.

[24] Amerikanou C, Kleftaki S A, Valsamidou E, Chroni E, Biagki T, Sigala D (2023). Food, dietary patterns, or is eating behavior to blame?analyzing the nutritional aspects of functional dyspepsia. Nutrients.

[25] Yamamoto Y, Furukawa S, Watanabe J, Kato A, Kusumoto K, Miyake T (2022). Association between eating behavior, frequency of meals, and functional dyspepsia in young Japanese population. J Neurogastroenterol Motil.

[26] Pepoyan A, Trchounian A (2009). Biophysics, molecular and cellular biology of probiotic activity of bacteria. Bacterial membranes.

[27] Pepoyan A, Mikayelyan M, Grigoryan H, Stepanyan L, Mirzabekyan S, Malkhasyan L (2024). Challenges for heat stress:Intestinal culturable bacteria of Lohmann Brown chickens. Res Vet Sci.

[28] Pepoyan E, Marotta F, Manvelyan A, Galstyan A, Stepanyan L, Grigoryan H (2024). Placebo-resistant gut bacteria:*Akkermansia muciniphila* spp. and familial Mediterranean fever disease. Front Cell Infect Microbiol.

[29] Harutyunyan N, Stepanyan L, Balayan M, Manvelyan A, Pepoyan E, Tsaturyan V (2025). Preliminary evidence for sex-specific trends in probiotic modulation of gut Saccharibacteria in familial Mediterranean fever patients:Effects of *Lactobacillus acidophilus* INMIA 9602 Er 317/402 and *Escherichia coli* M-17. Int J Mol Sci.

[30] Askari A, Pepoyan A, Parsaeimehr A (2012). Salt tolerance of genetic modified potato (Solanum tuberosum cv. Agria) by expression of a bacterial *mtlD* gene. Adv Agric Bot.

[31] Galstyan L, Tsaturyan V, Pepoyan A (2018). Efficiency of pre- and probiotic therapy for the management of periodic disease and hypoxic ischemic encephalopathy of newborns:*NLRP3* inflammasome. PARMA.

[32] Manvelyan A, Balayan M, Miralimova S, Chistyakov V, Pepoyan A (2023). Biofilm formation and auto-aggregation abilities of novel targeted aqua-probiotics. Funct Food Health Dis.

[33] Rastmanesh R, Isacco C G, Vellingiri B, Pepoyan A, Marotta F, Tekin I (2025). Potassium-rich, gluten-free diets for patients with Sjögren's syndrome:a hypothesis. Endocr Metab Immune Disord Drug Targets.

[34] Pepoyan A, Tsaturyan V, Manukyan V, Egorov I, Ilina L, Ronzhin A, Kostyaev A (2023). Novel probiotic *Lactiplantibacillus plantarum* str ZPZ as a possible candidate for “One Health”probiotic. Agriculture Digitalization and Organic Production.

[35] Pepoyan A, Chikindas M L (2025). Comparative biosafety and efficacy of *Pseudomonas fluorescens* PFS and *Lactiplantibacillus plantarum* ZPZ against *Ralstonia solanacearum*. Sci Rep.

[36] Conference of Research Workers in Animal Diseases (2025). Proceedings of the 2025 Conference of Research Workers in Animal Diseases, January 18–21, 2025, Chicago, IL, USA. Conference program and abstracts. CRWAD.

[37] Mini review article (2022). Planetary health perspectives on environmental and biological confounders affecting population health. Front Public Health.

[38] Grabow M, Ullmann W, Landgraf C, Sollmann R, Scholz C, Nathan R (2024). Sick without signs:subclinical infections reduce local movements, alter habitat selection, and cause demographic shifts. Commun Biol.

[39] Ramírez-Castillo F Y, Loera-Muro A, Jacques M, Garneau P, Avelar-González F J, Harel J (2015). Waterborne pathogens:detection methods and challenges. Pathogens.

[40] Butterworth N J, Heffernan L, Hall M D (2024). Is there a sicker sex?Dose relationships modify male–female differences in infection prevalence. Proc Biol Sci.

[41] Burdick Sanchez N C, Broadway P R, Carroll J A (2022). Sexual dimorphic innate immune response to a viral–bacterial respiratory disease challenge in beef calves. Vet Sci.

